# Effect of Graphic Warning Labels on Cigarette Pack–Hiding Behavior Among Smokers: The CASA Randomized Clinical Trial

**DOI:** 10.1001/jamanetworkopen.2022.14242

**Published:** 2022-06-02

**Authors:** John P. Pierce, Sheila Kealey, Eric C. Leas, Kim Pulvers, Matthew D. Stone, Jesica Oratowski, Elizabeth Brighton, Adriana Villaseñor, David R. Strong

**Affiliations:** 1 Cancer Control Program, Moores Cancer Center, University of California, San Diego, La Jolla; 2 Herbert Wertheim School of Public Health and Human Longevity Science, University of California, San Diego, San Diego; 3 Department of Psychology, California State University, San Marcos; 4 Department of Epidemiology, Public Health Services, San Diego County, San Diego, California

## Abstract

**Importance:**

The inclusion of graphic warning labels (GWLs) on cigarette packs is recommended for tobacco control but has not yet been implemented in the US. It is unknown whether and to what extent the inclusion of GWLs on cigarette packs affects smokers’ willingness to display the packs in public.

**Objective:**

To determine whether the inclusion of GWLs on cigarette packs affects pack-hiding behavior among smokers in social settings.

**Design, Setting, and Participants:**

This community-based randomized clinical trial assessed smokers’ real-world experience of using cigarettes repackaged to include GWLs (GWL packs) compared with standard US packs and blank packs over a 3-month intervention period with 12 months of follow-up between September 6, 2016, and December 3, 2019. The study included 357 participants aged 21 to 65 years from San Diego County, California, who smoked 5 or more cigarettes per day, were not actively planning to quit smoking, were not pregnant, and had no unstable medical conditions. Participants purchased and received cigarette packs through the study website.

**Interventions:**

During the 1-month run-in period, participants received their usual US cigarette packs. During the 3-month intervention period, participants were randomized to receive GWL packs (study-manufactured packs with 3 rotating images under license from the Commonwealth of Australia; GWL pack group), blank packs (study-manufactured packs devoid of industry marketing imagery; blank pack group), or standard US packs (US pack group).

**Main Outcomes and Measures:**

Pack hiding was queried daily (with participants reporting behavior within the last 4 hours) and weekly via interactive text messages during the 1-month run-in and intervention periods. Self-reported smoking behavior was biochemically validated.

**Results:**

Among 357 enrolled participants, the mean (SD) age was 39.3 (11.8) years; 195 participants (54.6%) were female, 40 (11.2%) were Hispanic, 243 (68.1%) were non-Hispanic White, and 74 (20.7%) were of other non-Hispanic races (including American Indian or Alaska Native, Asian or Pacific Islander, Black or African American, or multiracial). A total of 18 987 cigarette packs were purchased and delivered during the run-in and intervention periods. Daily querying showed that the inclusion of GWLs on cigarette packs increased the percentage of smokers who hid their packs at least some of the time from 41.3% (95% CI, 39.6%-43.0%) during the run-in period to 57.1% (95% CI, 55.9%-58.1%) by the end of the intervention period. In the postintervention period, returning to standard US packs reduced pack-hiding behavior to the levels observed during the run-in period. Pack hiding remained at run-in levels for both the blank pack group (35.2%; 95% CI, 33.6%-36.8%) and the US pack group (41.4%; 95% CI, 39.7%-43.1%]) throughout the study. Although even participants in the GWL group with the lowest prestudy tendency to conceal score (ie, 1) had a mean (SE) probability of pack hiding during the intervention of 0.84 (0.02), this group’s probability of pack hiding decreased to a mean (SE) of 0.43 (0.03) after intervention. When social reactions to packs were queried at the end of the study, the modal response from participants in the GWL pack group was observers’ aversive reactions to the packs, whereas the modal response from participants in the blank pack group was observers’ positive interest in the study. Neither smoking prevalence nor consumption differed by group at any point in the study.

**Conclusions and Relevance:**

In this randomized clinical trial, receiving cigarettes in GWL packs vs blank packs increased pack-hiding behavior in social settings, which may be associated with aversive reactions from observers. However, 12-month smoking behavior did not change.

**Trial Registration:**

ClinicalTrials.gov Identifier: NCT02676193

## Key Points

**Question:**

Do graphic warning labels (GWLs) on cigarette packs affect pack-hiding behavior among smokers in social settings?

**Findings:**

In this randomized clinical trial of 357 smokers, repackaging cigarettes with GWLs significantly increased the percentage of smokers who reported pack-hiding behavior at least some of the time from 41% to 57% through the intervention period. However, the inclusion of GWLs on cigarette packs had no effect on smoking behavior.

**Meaning:**

This study’s findings suggest that including GWLs on cigarette packs increase pack-hiding behavior among smokers, which may be in response to aversive social reactions to displaying GWL packs.

## Teaser

This randomized clinical trial assesses whether the inclusion of graphic warning labels on cigarette packs affects pack-hiding behavior among smokers in social settings.

## Introduction

Smokers have historically accessed their cigarette packs frequently in public settings so that their packs were commonly on display, creating a unique marketing opportunity for cigarette brands. This high degree of visibility can reinforce the social identity of the cigarette brand,^1^ strengthening the impression that smoking is acceptable in many social contexts.^2,3^ To remove this pervasive marketing opportunity and reduce the appeal of tobacco products, the World Health Organization’s Framework Convention on Tobacco Control recommends that countries mandate removal of industry marketing imagery from tobacco packaging with replacement by large graphic warning labels (GWLs) displaying the health consequences of smoking.^4,5^ These measures have not yet been implemented in the US.

There is considerable evidence from cross-cultural, cross-sectional surveys revealing that normative beliefs about smoking and the willingness of nonsmokers to take action to prevent exposure to secondhand smoke are associated with the prevalence of smoking behaviors.^6,7,8,9,10,11,12,13^ When visible to others, cigarette packs with large GWLs could increase the smoking-related stigma of smokers,^12,14^ who may react by hiding their cigarette packs from public view. Community observation reports after implementation of GWL packs in different countries have suggested that a major effect of GWLs might be a reduction in smokers’ willingness to display cigarette packs in social settings, such as cafes,^15,16^ and that this decrease might be associated with lower perceived social acceptability of smoking over the long term, especially among teens.^17,18^

In this randomized clinical trial, we introduced cigarette packs with GWLs (GWL packs) to smokers in San Diego, California, and measured aversive reactions to the GWL images.^19^ We then randomized participants to receive cigarettes repackaged as GWL packs (GWL pack group), as blank packs (blank pack group), or as standard US packs (US pack group) for an intervention period of 3 months. A previous study reported that GWL packs decreased smokers’ positive perceptions of their cigarettes, increased their perceptions of harm from smoking, and altered their cognition regarding quitting but did not lead to short-term changes in smoking behavior.^20^

This article reports the results of additional primary aims to investigate whether the inclusion of GWLs on cigarette packs resulted in decreased willingness to display the packs in public during and after the intervention period and whether long-term changes in smoking behavior occurred. We assessed whether the use of different cigarette pack designs led to an increase in pack-hiding behavior in social settings and whether pack-hiding behavior returned to baseline levels after the intervention period. We hypothesized that over the 3-month intervention, pack-hiding behavior would increase only among smokers randomized to receive GWL packs and only during the time they had GWL packs. We also investigated which smokers were most likely to change their pack-hiding behavior and examined whether long-term changes in smoking behavior occurred.

## Methods

Between September 6, 2016, and December 3, 2019, we recruited smokers from San Diego County, California, to participate in the Effect of Packaging on Smoking Perceptions and Behavior (CASA) randomized clinical trial, with follow-up completed on December 9, 2020.^17^ The CASA trial protocol is available in Supplement 1. The study was approved by the institutional review boards of the University of California, San Diego, and California State University, San Marcos. Participants provided written informed consent. This study followed the Consolidated Standards of Reporting Trials (CONSORT) reporting guideline for randomized clinical trials.^21^

Our enrollment target was 150 participants per group based on statistical power greater than 0.85 (α = .025) for medium between-group effects (Cohen *d*≥0.25), assuming a 20% withdrawal rate. Eligible participants were aged 21 to 65 years, smoked 5 or more cigarettes per day, were not actively planning to quit smoking, had no unstable medical conditions, and were not pregnant. The study design (eFigure 1 in Supplement 2) included a 4-week run-in period to assess participant adherence to study measures (required for randomization).

**Figure 1.**
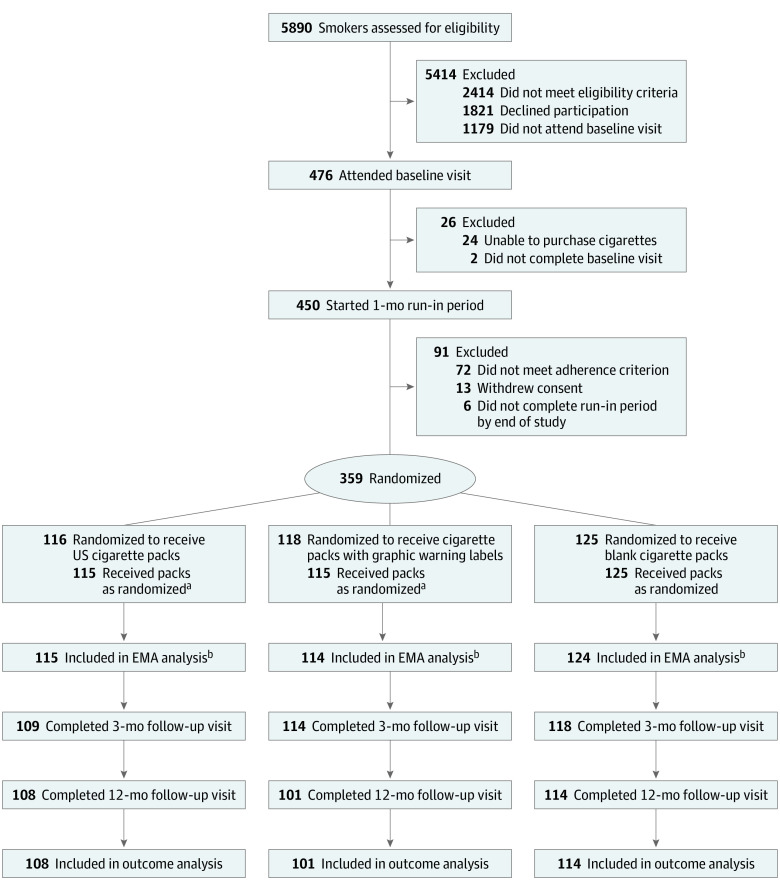
Flow Diagram for the CASA Randomized Clinical Trial Participants purchased their usual packs through the study website. During the run-in period, all received their usual US packs. During the intervention period, their cigarettes were repackaged according to study group. CASA indicates Effect of Packaging on Smoking Perceptions and Behavior randomized clinical trial. ^a^Two participants were excluded shortly after randomization (with approval from the data safety monitoring board) because they resided in the same house and had been randomized to different groups (1 randomized to receive US packs and 1 randomized to receive packs with graphic warning labels). ^b^Participants included in the ecological momentary assessment (EMA) analysis completed interactive daily text messages to evaluate short-term smoking behavior and cognition.

A computerized urn randomization procedure^22^ was used to randomize 359 participants to receive GWL cigarette packs, blank cigarette packs, or standard US cigarette packs. Groups were stratified based on 3 binary variables (age, sex, and nicotine dependence) using a 1:1:1 ratio, with assessment staff and investigators blinded to the randomization process (Figure 1). Two participants were excluded after randomization because they resided in the same house and had been randomized to different groups. Participants purchased cigarettes from the study website in both the run-in period (during which standard US packs were available for purchase) and the 3-month intervention period (during which study packs based on randomization group were available for purchase), and cigarette packs were delivered to participants by courier.

### Study Packs

For the 3-month intervention period, we manufactured 4 different cigarette packs. Three of those packs included GWLs featuring 3 rotating images (foot gangrene, neonatal baby, and throat stoma) under license from the Commonwealth of Australia (eFigure 2 in Supplement 2). The fourth pack (blank) had no industry marketing imagery and was colored to match the Australian GWL packs; these blank packs included the standard warning message from the US Surgeon General. The US pack group continued to receive their usual commercial packs throughout the study.

### Measurement

#### Smoking Behavior

At baseline and 12 months, participants completed a questionnaire including the following queries: (1) “On how many of the past 30 days did you smoke cigarettes?” (2) “During the past 30 days, on the days that you did smoke, about how many cigarettes did you usually smoke per day?” and (3) “Have you tried to quit smoking intentionally in the last 30 days?” Saliva samples were collected and analyzed for cotinine levels.

#### Pack-Hiding Behavior

Two interactive text messaging measures of pack-hiding behavior were conducted (1 daily and 1 weekly). The daily measure (during the run-in and intervention periods only) sent text messages to participants in both late morning and afternoon or evening and started with a query about the number of cigarettes smoked in the previous 4 hours. Participants were then asked, “In the last 4 hours, did you place the pack so that others would not see it?” Because smoking is not always done in the company of others, we used the percentage of participants reporting that they hid their pack at least some of the time as the indicator for this outcome. The mean of the twice-daily responses was calculated to provide a single probability of hiding packs at least some of the time. We used the 4-week run-in period during which participants received standard US packs to establish a baseline. To reduce participant burden after completion of the intervention, we also included a weekly interactive text message that measured pack hiding across the whole study year by asking, “In the past week, how often did you keep the pack out of view?” Response options to these questions were never (coded as 1), some of the time (coded as 2), most of the time (coded as 3), and always (also coded as 3).

#### Study Covariates

The prestudy questionnaire comprised items about sociodemographic characteristics (including age, sex, race and ethnicity, educational level, and household income^17^) and the Fagerström Test for Nicotine Dependence (score range, 0-10 points, with higher scores indicating greater intensity of nicotine dependence).^23^ Based on previous work,^24,25^ pack branding appeal was assessed through responses to the following statement: “The design on the brand of cigarettes I currently smoke is…stylish, fashionable, cool, high quality, attractive, appealing.” Respondents used a 6-point Likert scale to indicate their extent of agreement with each adjective, with 1 indicating strongly disagree and 6 indicating strongly agree. The item had strong scalability (mean [SE] Mokken H coefficient, 0.68 [0.02])^26^ and excellent internal consistency (α = .92).

A prestudy tendency to conceal scale assessed the following 4 statements about participants’ behavior within the past 30 days using a 3-point Likert scale for each item (with 1 indicating never, 2 indicating some of the time, and 3 indicating most of the time or always): (1) “I tried to keep my cigarette packs hidden, where people couldn’t see them,” (2) “I tried to avoid smoking in public places (like at a party or outside restaurant),” (3) “I tried to avoid smoking with close friends, coworkers or family members that I’ve smoked with before,” and (4) “I tried to hide my smoking from my close friends or family members.” This scale had a range for mean scores of 1 to 3. The scale had good scalability (mean [SE] Mokken H coefficient, 0.49 [0.03]) and good internal consistency (α = .74).

### Recalled Reactions to the Study

At the last study visit, participants completed an open-ended online questionnaire about the benefits and challenges of study participation. Both the GWL pack and the blank pack groups were asked to answer the following open-ended questions in their own words: (1) “Looking back, did you ever experience any unusual circumstances while carrying the study packs of cigarettes?” and (2) “Did you ever encounter any difficult reactions from family members and/or friends when you opened the box of cigarettes?” Two authors (J.O. and E.B.) independently reviewed a subset of responses and agreed on major themes. The full set was then coded and consensus obtained on discrepancies.

### Statistical Analysis

Analyses included all available data from each randomized group and were conducted using R software, version 3.6.1 (R Foundation for Statistical Computing).^27^ For missing data (9% of weekly assessments and 15% of daily assessments), we imputed 40 data sets, and the Rubin rule^28,29^ was used to pool model estimates and obtain SEs using the Amelia package of R software. For the measure of daily pack-hiding behavior, we plotted the proportion of each study group who indicated at least some pack-hiding behavior during the run-in and intervention periods. For the weekly measure of pack-hiding behavior, we plotted the difference from the baseline measure over time in the proportion of each group who reported that they hid their pack at least some of the time (we calculated the mean of run-in assessments to provide a single baseline estimate). Each plot included a nonparametric local polynomial smoothing line with a span of 0.75 (using the loess function in the stats package of R software) to evaluate the nonlinear form of patterns over time before regression model fitting.

To compare patterns across study groups, we used the full scale for each rating and applied an ordered logistic model with mixed effects and a cumulative link,^30^ with a knot at study week 8 (week 4 of intervention) to identify any early intervention response observed in the postintervention analysis. These models estimated the cumulative mean probability of a rating being higher than each category of response (ie, a threshold). No model structure was imposed on the distances between thresholds for increasing categories of pack hiding. Changes in the odds (logit) of endorsing categories that reflected more pack hiding were modeled as a linear pattern over time, with adjustment for baseline frequency of pack hiding, slope (group and slope interaction) for each group, and a term reflecting whether observations were recorded during the intervention or during the 8-month follow-up period. A planned interaction between baseline pack-hiding behavior and treatment arm was included if supported by a likelihood ratio test. All tests of interactions included lower-order terms, and differences were reported using model estimates of the mean probability of pack hiding (eTable 2 in Supplement 2). All models were adjusted for age, sex, race and ethnicity, baseline pack branding appeal, and baseline nicotine dependence levels. The threshold for statistical significance was 2-tailed *P* = .05.

## Results

### Characteristics of Population

Among 357 participants, the mean (SD) age was 39.3 (11.8) years; 195 participants (54.6%) were female, and 162 (45.4%) were male (eTable 1 in Supplement 2). A total of 40 participants (11.2%) were Hispanic, 243 (68.1%) were non-Hispanic White, and 74 (20.7%) were of other non-Hispanic races (including American Indian or Alaska Native, Asian or Pacific Islander, Black or African American, or multiracial). Overall, 148 participants (41.5%) had a college degree, and 143 (40.1%) had household income less than $50 000 per year.

A total of 117 participants were randomized to the GWL pack group, 125 to the blank pack group, and 115 to the standard US pack group. Randomization achieved group equivalence in demographic characteristics and baseline smoking variables, including the number of cigarettes smoked per day within the past 7 days (GWL pack group: mean [SD], 11.9 [8.7] cigarettes; blank pack group: mean [SD], 12.9 [8.9] cigarettes; US pack group: mean [SD], 13.0 [10.2] cigarettes), serious attempts to quit within the past year (GWL pack group: 48 participants [41.0%]; blank pack group: 55 participants [44.0%]; US pack group: 53 participants [46.1%]), nicotine dependence based on Fagerström Test for Nicotine Dependence score (GWL pack group: mean [SD], 3.7 [2.3] points; blank pack group: 3.9 [2.3] points; US pack group: 3.8 [2.3] points), current cigarette brand (Marlboro, Camel, or American Spirit; GWL pack group: 100 participants [85.5%]; blank pack group: 109 participants [87.2%]; US pack group: 98 participants [85.2%]), loyalty to current brand (GWL pack group: 88 participants [75.2%]; blank pack group: 97 participants [77.6%]; US pack group: 84 participants [73.0%]), and pack branding appeal based on 6-point Likert scale (GWL pack group: mean [SD], 3.9 [1.1] points; blank pack group: mean [SD], 3.7 [1.2] points; US pack group: mean [SD], 3.5 [1.3] points).

Over the run-in and intervention periods, 18 987 cigarette packs were delivered to participants via courier. By December 9, 2020, 108 participants (93.9%) randomized to the US pack group, 101 (86.3%) randomized to the GWL pack group, and 114 (91.2%) randomized to the blank pack group completed the final study visit. There were no differences by study group in response to either the daily or weekly assessments, and no adverse events were reported by any participant at any time during the study.

### Pack-Hiding Behavior in Daily Assessments

**Figure 2.**
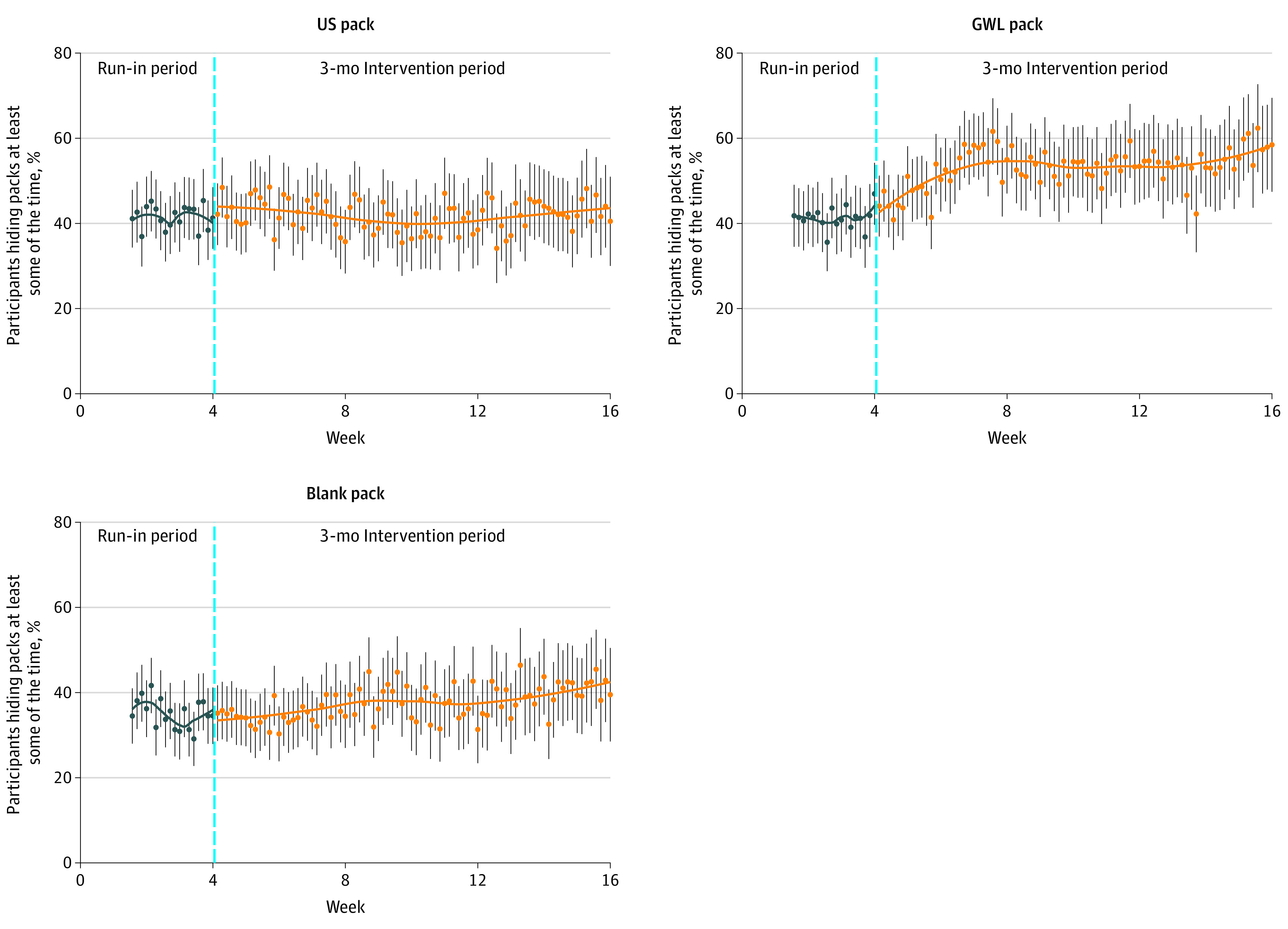
Daily Mean Percentage of Participants Who Hid Cigarette Packs at Least Some of the Time Assessed by daily interactive text messaging during run-in and 3-month intervention periods. Dots represent mean daily values with 95% CIs (indicated by whiskers). A nonparametric local polynomial regression model with smoothing span of 0.75 was plotted to describe the nonlinear trend. Blank cigarette packs had no industry marketing imagery, graphic warning label (GWL) packs featured 1 of 3 rotating images, and US packs were standard packs that participants usually purchased.

Throughout the run-in and intervention periods, in response to daily text messages, respondents indicated smoking a median of 3 cigarettes (range, 0-12 cigarettes) over the previous 4 hours. We plotted the mean daily percentage of each group who reported hiding their packs at least some of the time (Figure 2). During the run-in, across the 3 study groups, mean pack hiding was 39.5% (95% CI, 38.6%-40.4%). In the US pack group, during the run-in or baseline period, 41.4% (95% CI, 39.7%-43.1%) reported hiding their packs at least some of the time; this level of pack hiding did not change throughout the intervention period (week 8: 43.6% [95% CI, 41.7%-45.3%]; week 12: 43.2% [95% CI, 41.0%-45.4%]). The baseline for the GWL pack group (41.3%; 95% CI, 39.6%-43.0%) was not different from that of the US pack group. However, receiving cigarettes in GWL packs significantly increased participants’ reported pack hiding. Pack hiding increased gradually over the first 4 weeks of the intervention (week 8: 57.1%; 95% CI, 55.9%-58.1%) and remained at this level through the end of the intervention (week 12: 59.0%; 95% CI, 57.6%-61.2%). In the blank pack group, the prestudy tendency to conceal packs (35.2%; 95% CI, 33.6%-36.8%) was significantly lower than pack-hiding behavior in either of the other 2 study groups; no significant change in this level was observed throughout the intervention (week 8: 36.9% [95% CI, 35.2%-38.9%]; week 12: 41.5% [95% CI, 38.8%-44.2%]).

In our model of the probability of pack hiding, no significant effects were observed for sociodemographic characteristics, baseline nicotine dependence, or baseline pack branding appeal (Table 1). The model showed that the probability of pack hiding during the intervention was strongly influenced by the pattern observed during the run-in period. Prestudy tendency to conceal was also significantly associated with probability of pack-hiding behavior during the intervention (*z* score = 2.5; *P* = .01). A sensitivity analysis showed that including both prestudy tendency to conceal packs and prestudy pack branding appeal improved the overall model fit (χ^2^ likelihood ratio with vs without both variables = 6.03; *df* = 2; *P* = .049). This full model confirmed that no increase in pack hiding occurred in either the US pack group (probability in week 4: 0.34; 95% CI, 0.19-0.54) or the blank pack group (probability in week 4: 0.24; 95% CI, 0.12-0.42), whereas a significant increase occurred in the GWL pack group (mean probability in week 4: 0.72; 95% CI, 0.54-0.84) (eFigure 3 in Supplement 2). Thus, the difference in probability between GWL and US groups after 4 weeks of intervention was 0.38 (95% CI, 0.72-0.34).

**Table 1.** Ordinal Logistic Model of Daily Assessments of Change in Cigarette Pack-Hiding Behavior^a^

Variable	β (SE)	*z* Score	*P* value
Age	0 (0.01)	−0.29	.77
Sex			
Male	1 [Reference]	−0.44	.66
Female	−0.10 (0.22)		
Race and ethnicity			
Hispanic	−0.10 (0.35)	−0.28	.78
Non-Hispanic White	1 [Reference]	NA	NA
Non-Hispanic other race^b^	−0.06 (0.26)	−0.24	.81
Nicotine dependence	−0.01 (0.05)	−0.17	.87
Prestudy pack branding appeal	0.12 (0.09)	1.27	.20
Prestudy tendency to conceal scale	0.50 (0.20)	2.50	.01
Pack hiding during 1-mo run-in period	3.88 (0.18)	21.79	<.001
Study group effect			
US pack	1 [Reference]	NA	NA
GWL pack	−0.31 (0.34)	−0.94	.35
Blank pack	0.10 (0.34)	0.30	.77
Intervention timing effect			
Early (weeks 1-4)	−0.05 (0.23)	−0.21	.84
Later (weeks 5-12)	−0.07 (0.21)	−0.32	.75
Early effects (weeks 1-4)			
US pack	1 [Reference]	NA	NA
GWL pack	1.31 (0.33)	3.91	<.001
Blank pack	−0.35 (0.35)	−0.99	.32
Later effects (weeks 5-12)			
US pack	1 [Reference]	NA	NA
GWL pack	1.34 (0.30)	4.43	<.001
Blank pack	0.28 (0.34)	0.81	.42

Abbreviations: GWL, graphic warning label; NA, not applicable.

^a^ Missing data were imputed (40 imputed data sets), and the Rubin rule was used to pool estimates using the Amelia package in R software, version 3.6.1 (R Foundation for Statistical Computing).

^b^ Participants who were of non-Hispanic other race comprised 21% of the study sample and included participants who were American Indian or Alaska Native, Asian, Black or African American, Native Hawaiian or Pacific Islander, or multiracial.

### Postintervention Changes in Pack-Hiding Probability

During the intervention period, weekly assessments were correlated with daily assessments (*r *> 0.74), and the pattern of pack hiding observed in the weekly assessment data was similar to that of daily assessment data (week 8: mean [SE] change in pack hiding, −5.5% [0.05%] in the US pack group, 6.6% [0.05%] in the blank pack group, and 15.1% [0.05%] in the GWL pack group) (eFigure 4 in Supplement 2). At 4 weeks, the difference in probability between GWL and US groups was 0.16, which was 41.3% lower than the assessment using the daily measure. Given the difference in prestudy tendency to conceal across groups using this weekly measure, we modeled the percentage difference from baseline for each study group (eTable 2 in Supplement 2). The GWL intervention increased the probability of pack hiding some of the time by 15.1% (95% CI, 5.7%-24.6%), and this increase was maintained until the end of the intervention (14.1%; 95% CI, 4.4%-23.9%). As with the daily measure, there was no intervention effect in either the blank pack or US pack groups. At week 16 (1 month after completion of the intervention), pack hiding by the GWL pack group had decreased to the extent that it was no different from baseline levels (−0.4%; 95% CI, −12.1% to 11.3%).

### Prestudy Tendency to Conceal and Pack Hiding at Intervention and Follow-up

**Figure 3.**
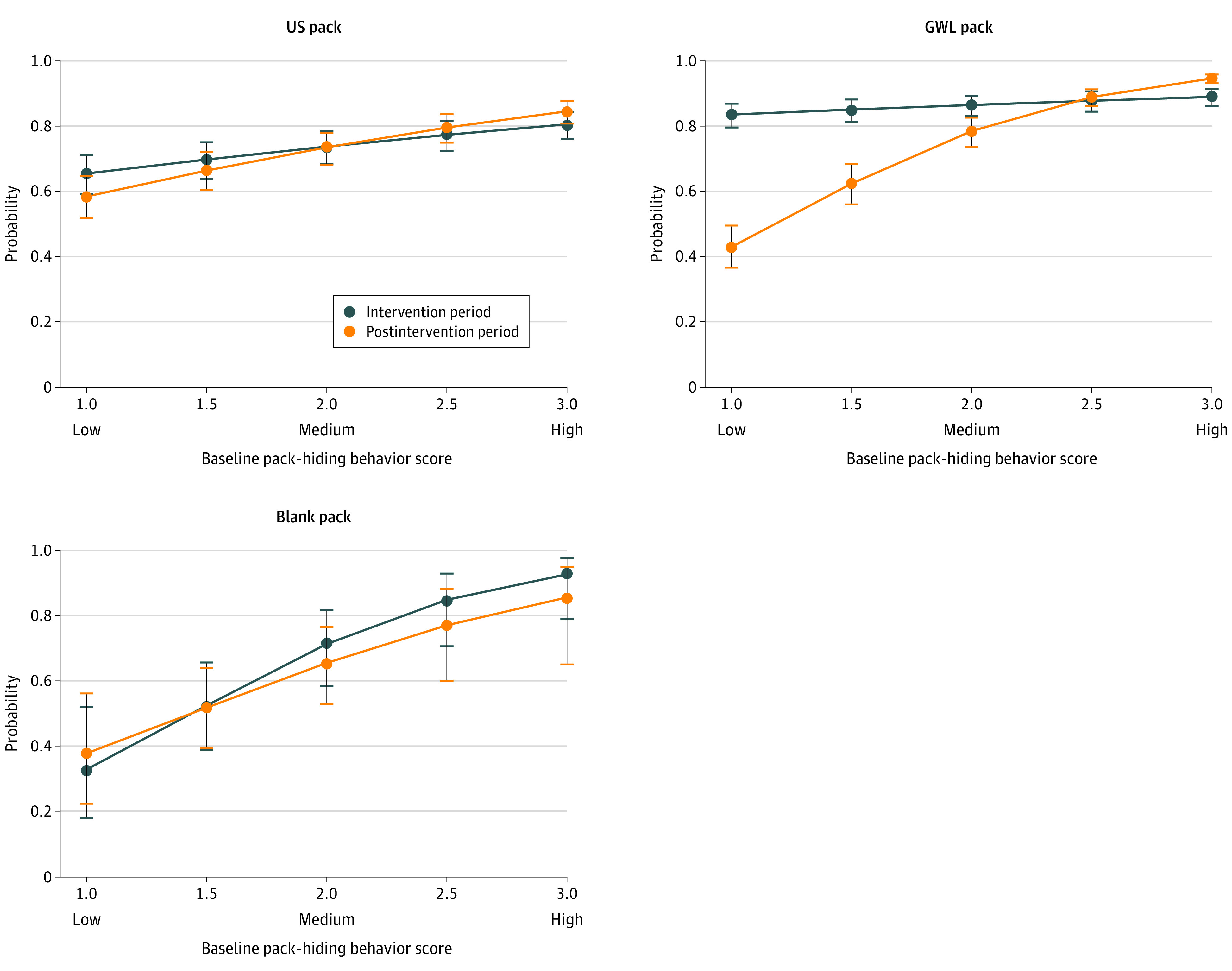
Prestudy Tendency to Conceal Packs and Differences in Probability of Pack-Hiding Behavior During and After the Intervention by Study Group Plotted probabilities are from adjusted ordinal regression models and include 95% CIs (indicated by whiskers) for each model-based estimate. Prestudy tendency to conceal reflects mean responses to 4 questionnaire items, each scored using a 3-point Likert scale, with 1 indicating low pack hiding (packs were never hidden), 2 indicating medium pack hiding (packs were hidden some of the time), and 3 indicating high pack hiding (packs were hidden most or all of the time). Blank cigarette packs had no industry marketing imagery, graphic warning label (GWL) packs featured 1 of 3 rotating images, and US packs were standard packs that participants usually purchased.

We plotted the probabilities of pack hiding at least some of the time (based on adjusted ordinal regression models of weekly assessments shown in eTable 2 in Supplement 2) among participants by quintile of prestudy tendency to conceal scale score (all of the lowest quintiles had a score of 1 on this scale) (Figure 3). In the groups receiving standard US packs and blank packs, pack-hiding behavior increased as quintile increased, regardless of whether the measure was assessed in the intervention or postintervention period. For example, those in the US pack group with a score of 1 on this scale had a mean probability of 0.66 (0.03), and those with a score of 2 had a mean probability of 0.74 (0.30) during the intervention. A different effect was observed in the GWL pack group, with all quintiles showing a high probability of hiding packs at least some of the time during the intervention (a score of 1 had a mean [SE] probability of 0.84 [0.02] and a score of 2 had a mean [SE] probability of 0.87 [0.02] during the intervention). Participants with lower prestudy tendency to conceal scores showed a significant decrease in their probability of pack hiding after the intervention. Those with a score of 1 decreased from a mean (SE) probability of 0.84 (0.02) during intervention to a mean (SE) probability of 0.43 (0.03) after the intervention. This finding was supported in the model with significant 3-way interaction terms (prestudy tendency to conceal, treatment arm, and intervention and postintervention measure; GWL pack interaction: β [SE], 0.78 [0.23]; *z* = 3.43; *P* = .001; blank pack interaction: β [SE], −0.48 [0.24]; *z* = −1.98; *P* = .048) (eTable 2 in Supplement 2).

### Change in Smoking Behavior

We used regression models to compare smoking behavior among groups at the end of the study, with adjustment for planned covariates and corresponding baseline values (Table 2). All estimates decreased in all groups. At 12 months, we observed no significant differences in the odds of daily smoking between the GWL pack and US pack groups (odds ratio [OR], 1.12; 95% CI, 0.58-2.17) or the blank pack and US pack groups (OR, 0.86; 95% CI, 0.47-1.59). No significant differences in the mean number of smoking days within the past 30 days were found between the GWL pack and US pack groups (β [SE], 0.46 [1.45]; *P* = .75) or the blank pack and US pack groups (β [SE], −0.16 [0.39]; *P* = .91). In addition, no significant differences in the mean number of cigarettes smoked per day were found between the GWL pack and US pack groups (β [SE], 0.25 [0.69]; *P* = .72) or the blank pack and US pack groups (β [SE], −0.18 [0.66]; *P* = .79). Saliva cotinine measures validated a lack of difference between study groups at each measurement point.

**Table 2.** Measures of Smoking Behavior From Study Questionnaires at Baseline and End of Study

Measure	Estimate (95% CI)	
	Baseline^a^	End of study (12 mo)^b^
Percentage of current daily smokers		
US pack	98 (96-100)	76 (66-83)
GWL pack	97 (95-100)	78 (68-85)
Blank pack	100 (100-100)	73 (64-80)
Mean frequency of smoking in past 30 d		
US pack	29.6 (29.3-29.9)	24.2 (22.2-26.2)
GWL pack	29.3 (28.8-29.8)	24.7 (22.6-26.7)
Blank pack	29.5 (29.2-29.9)	24.0 (22.1-25.9)
Mean cigarettes smoked/d		
US pack	11.8 (10.8-12.9)	9.0 (8.0-9.0)
GWL pack	11.2 (10.1-12.3)	9.2 (8.2-10.2)
Blank pack	12.0 (10.9-13.1)	8.8 (7.9-9.7)

Abbreviation: GWL, graphic warning label.

^a^ Baseline questionnaire was completed during the first study visit before the start of the run-in period (approximately 1 month before randomization).

^b^ End-of-study questionnaire was completed approximately 8 months after completion of the intervention period.

### Perceived Benefits and Challenges

At the end of the study, 279 participants (85 [73.3%] in the US pack group, 85 [72.0%] in the GWL pack group, and 109 [87.2%] in the blank pack group) had completed the open-ended online questionnaire regarding the benefits and challenges of study participation. Among those in the GWL pack group, the highest coded response (26 participants [30.6%]) to the unusual circumstances experienced during the study was that family, friends, and others in close proximity had an aversive reaction to the pack; 21 participants (24.7%) reported that family and friends expressed interest in the study, with 11 participants (12.9%) indicating that this interest resulted in increased pressure to quit smoking. Among the blank pack group, the highest coded response (38 participants [34.9%]) was that the pack was a conversation starter, with family, friends, and others expressing interest in the study.

## Discussion

In this randomized clinical trial, during the 1-month run-in period, 39.5% of smokers reported hiding their cigarette packs from observers at least some of the time. Repackaging cigarettes as GWL packs significantly increased the proportion of participants who hid their packs from 41.3% to 57.1% by intervention week 4, and this increase of 38.3% was maintained throughout the remainder of the 3-month intervention. When the GWL pack group returned to their usual US cigarette packs, pack-hiding behavior returned to levels observed during the run-in period. This pack-hiding effect was specific to participants who received GWL packs. Those who received either blank packs devoid of industry marketing imagery or standard US packs did not increase their pack-hiding behavior during the study. However, repackaging cigarettes into GWL packs was not sufficient to change smoking behavior.

During the intervention, the probability of pack hiding in the GWL pack group was similarly high across all quintiles of the prestudy tendency to conceal scale; however, the postintervention decrease in pack hiding was only significant among those in the lower 2 quintiles of the prestudy tendency to conceal scale. Thus, receiving cigarettes in GWL packs increased pack hiding among those with the lowest tendency to conceal their packs at baseline. When offered the opportunity to provide open-ended comments on unusual circumstances experienced during the intervention, the modal response from the GWL pack group was the aversive reaction of observers, whereas in the blank pack group, the modal response was observer expressions of interest in the study.

When first exposed to the different package designs before randomization, participants demonstrated considerable aversion to the GWL packs compared with the blank and standard US packs used in the other study arms,^19^ and these reactions were confirmed by an online willingness to pay study that explored purchase choices when multiple different packaging options were varied.^31^ If this aversive reaction prompted smokers in the GWL pack group to hide their packs, we might expect an immediate increase in pack hiding after participants received their study packs. However, it took weeks to reach a stable level of pack hiding, which was then maintained throughout the intervention. This finding suggests an alternate hypothesis in which the display of the GWL packs may have led to aversive reactions among observers, and the cumulative effect of observers’ negative comments may have increased the probability of pack hiding among participants. The comments of participants in the GWL pack group at the end-of-study review suggested that this second hypothesis may be correct, particularly when contrasted with the positive social comments reported by participants in the blank pack group.

### Strengths and Limitations

This study has several strengths. The study was a randomized clinical trial in which cigarettes were repackaged as high-quality study-manufactured packs and delivered to participants, with baseline pack-hiding likelihood assessed over a 4-week run-in period. The twice-daily interactive text messages querying pack-hiding behavior over the past 4 hours (with participants reporting a mean of 3 cigarettes smoked) is a strength because the proximity of the measurement to the target behavior minimized recall bias. The extent of the GWL effect from the proximal daily measures was 41.3% higher than that of the more general weekly measure. Notably, both measures identified the same pattern of change in pack hiding. An additional strength was that self-reported smoking behavior was confirmed by saliva cotinine levels at all time points.

This study also has limitations. Participants were smokers residing in California, a state with low smoking prevalence and relatively high antismoking social norms.^7^ However, the first exposure to the GWL packs produced a strong aversion in the study population, and this reaction was also reflected in the debriefing at the end of the study, during which the GWL pack group reported a similar aversion among observers when the pack was displayed. It is not clear to what extent high antismoking norms in the general population could have affected this aversive response. One possibility is that observers from areas with high antismoking norms may be more likely to comment about the social acceptability of smoking. We did not interview people within the smokers’ social networks nor did we specifically ask all participants in the GWL pack group how observers reacted when they displayed their packs. Instead, we allowed them to use their own words to identify unusual circumstances experienced during the intervention. The intervention was completed before the onset of the COVID-19 pandemic; however, the final in-person visit had to be changed to a virtual visit for approximately 25% of participants due to pandemic restrictions. In addition, during follow-up, COVID-19 restrictions may have increased solo smoking among some participants, thereby reducing the need to hide their cigarette packs from others.

## Conclusions

This randomized clinical trial found that the introduction of cigarette GWL packs to California smokers led to significantly higher pack-hiding behavior, particularly among those who were less likely to hide their usual US packs before the study. Removal of the GWL packaging eliminated the pack-hiding effect, but removal of industry marketing imagery did not affect pack-hiding behavior. The pack-hiding effect appeared to be related to observer reactions. However, the inclusion of GWLs on cigarette packs alone was not sufficient to increase quitting behavior.

## Notes

Supplement 1.
Trial Protocol

Supplement 2. **eTable 1.** Sample Characteristics by Randomization Group **eTable 2.** Ordinal Logistic Model of Weekly Assessments of Cigarette Pack-Hiding Behavior During Repackaging Intervention and After Return to Purchasing Their Usual US Pack **eFigure 1.** CASA Study Design **eFigure 2.** Images of Cigarette Packs Manufactured for the CASA Randomized Clinical Trial **eFigure 3.** Probability of Sometimes or Often “Placing the Pack Where Others Would Not See It” During the Intervention Period by Study Group, With 95% CIs **eFigure 4.** Patterns in Pack-Hiding Behavior by Study Group Assessed Weekly During the 3-Month Intervention and 8-Month Follow-up Periods

Supplement 3.
Data Sharing Statement

## References

[1] Cohen JB. Playing to win: marketing and public policy at odds over Joe Camel. J Public Policy Mark. 2000;19(2):155-167. doi:10.1509/jppm.19.2.155.17123

[2] Lynch BS, Bonnie RJ, eds; Institute of Medicine (US) on Preventing Nicotine Addiction in Children and Youths. *Growing Up Tobacco Free: Preventing Nicotine Addiction in Children and Youths*. National Academies Press; 1994. 25144107

[3] Echeverría SE, Gundersen DA, Manderski MTB, Delnevo CD. Social norms and its correlates as a pathway to smoking among young Latino adults. Soc Sci Med. 2015;124:187-195. doi:10.1016/j.socscimed.2014.11.034 25461876 PMC4276427

[4] World Health Organization. Plain packaging of tobacco products: evidence, design and implementation. May 30, 2016. Accessed August 31, 2021. https://www.who.int/publications/i/item/9789241565226

[5] McNeill A, Gravely S, Hitchman SC, Bauld L, Hammond D, Hartmann-Boyce J. Tobacco packaging design for reducing tobacco use. Cochrane Database Syst Rev. 2017;4(4):CD011244. doi:10.1002/14651858.CD011244.pub2 28447363 PMC6478110

[6] Elder J, Rosbrook B, Choi W, Johnson M, Bal D, Pierce JP. Public objections to environmental tobacco smoke. Prev Med. 1992;21(6):701-709. doi:10.1016/0091-7435(92)90077-U 1438116

[7] Pierce JP, Shi Y, McMenamin SB, . Trends in lung cancer and cigarette smoking: California compared to the rest of the United States. Cancer Prev Res (Phila). 2019;12(1):3-12. doi:10.1158/1940-6207.CAPR-18-0341 30305281 PMC7389269

[8] Karasek D, Ahern J, Galea S. Social norms, collective efficacy, and smoking cessation in urban neighborhoods. Am J Public Health. 2012;102(2):343-351. doi:10.2105/AJPH.2011.300364 22390449 PMC3483989

[9] Jackson SE, Proudfoot H, Brown J, East K, Hitchman SC, Shahab L. Perceived non-smoking norms and motivation to stop smoking, quit attempts, and cessation: a cross-sectional study in England. Sci Rep. 2020;10(1):10487. doi:10.1038/s41598-020-67003-8 32591555 PMC7320183

[10] Schoenaker DAJM, Brennan E, Wakefield MA, Durkin SJ. Anti-smoking social norms are associated with increased cessation behaviours among lower and higher socioeconomic status smokers: a population-based cohort study. PLoS One. 2018;13(12):e0208950. doi:10.1371/journal.pone.0208950 30540825 PMC6291149

[11] Dunbar MS, Nicosia N, Kilmer B. Exposure to new smoking environments and individual-level cigarette smoking behavior: insights from exogenous assignment of military personnel. Soc Sci Med. 2021;280:113983. doi:10.1016/j.socscimed.2021.113983 34020313 PMC8223508

[12] Evans-Polce RJ, Castaldelli-Maia JM, Schomerus G, Evans-Lacko SE. The downside of tobacco control? smoking and self-stigma: a systematic review. Soc Sci Med. 2015;145:26-34. doi:10.1016/j.socscimed.2015.09.026 26439764 PMC4630105

[13] Pierce JP, Gilpin EA, Emery SL, . *Tobacco Control in California: Who’s Winning the War? An Evaluation of the Tobacco Control Program, 1989-1996**.* University of California, San Diego; 1998. Accessed September 14, 2021. https://escholarship.org/uc/item/8st44338

[14] Nyborg K, Anderies JM, Dannenberg A, . Social norms as solutions. Science. 2016;354(6308):42-43. doi:10.1126/science.aaf8317 27846488

[15] Brennan E, Bayly M, Scollo M, Zacher M, Wakefield MA. Observed smoking and tobacco pack display in Australian outdoor cafes 2 years after implementation of plain packaging. Eur J Public Health. 2018;28(4):702-707. doi:10.1093/eurpub/cky051 29596579

[16] Nee-Nee J, Sutherland K, Holland R, . Tobacco pack display at hospitality venues after the introduction of standardised tobacco packaging in New Zealand: a field observation study. BMJ Open. 2019;9(9):e027868. doi:10.1136/bmjopen-2018-027868 31494599 PMC6731942

[17] Pierce JP, Strong DR, Stone MD, . Real-world exposure to graphic warning labels on cigarette packages in US smokers: the CASA randomized trial protocol. Contemp Clin Trials. 2020;98:106152. doi:10.1016/j.cct.2020.106152 32966877 PMC7502239

[18] El-Khoury Lesueur F, Bolze C, Gomajee R, White V, Melchior M; DePICT Study Group. Plain tobacco packaging, increased graphic health warnings and adolescents’ perceptions and initiation of smoking: DePICT, a French nationwide study. Tob Control. 2019;28(e1):e31-e36. doi:10.1136/tobaccocontrol-2018-054573 30409812 PMC6824742

[19] Stone M, Strong D, Dimofte C, . Role of affective reactivity induced by cigarette packaging including graphic warning labels: the CASA study. Tob Control. Published online September 12, 2021. doi:10.1136/tobaccocontrol-2021-056650 34511408 PMC8917242

[20] Strong DR, Pierce JP, Pulvers K, . Effect of graphic warning labels on cigarette packs on US smokers’ cognitions and smoking behavior after 3 months: a randomized clinical trial. JAMA Netw Open. 2021;4(8):e2121387. doi:10.1001/jamanetworkopen.2021.21387 34347057 PMC8339936

[21] Schulz KF, Altman DG, Moher D; CONSORT Group. CONSORT 2010 statement: updated guidelines for reporting parallel group randomized trials. Ann Intern Med. 2010;152(11):726-732. doi:10.7326/0003-4819-152-11-201006010-00232 20335313

[22] Wei LJ, Lachin JM. Properties of the urn randomization in clinical trials. Control Clin Trials. 1988;9(4):345-364. doi:10.1016/0197-2456(88)90048-7 3203525

[23] Heatherton TF, Kozlowski LT, Frecker RC, Fagerström KO. The Fagerström test for nicotine dependence: a revision of the Fagerström Tolerance Questionnaire. Br J Addict. 1991;86(9):1119-1127. doi:10.1111/j.1360-0443.1991.tb01879.x 1932883

[24] Skaczkowski G, Durkin S, Kashima Y, Wakefield M. The effect of packaging, branding and labeling on the experience of unhealthy food and drink: a review. Appetite. 2016;99:219-234. doi:10.1016/j.appet.2016.01.022 26785316

[25] Wakefield M, Germain D, Durkin S, Hammond D, Goldberg M, Borland R. Do larger pictorial health warnings diminish the need for plain packaging of cigarettes? Addiction. 2012;107(6):1159-1167. doi:10.1111/j.1360-0443.2012.03774.x 22372966

[26] Mokken RJ. A Theory and Procedure of Scale Analysis: With Applications in Political Research. Reprint ed. De Gruyter Mouton; 2011. *Methods and Models in the Social Sciences*, Series 1.

[27] R Core Team. The R project for statistical computing. The R Foundation. 2021. Accessed September 1, 2021. https://www.R-project.org/

[28] Rubin DB. Multiple Imputation for Nonresponse in Surveys. John Wiley & Sons; 1987.

[29] Honaker J, King G, Blackwell M. Amelia II: a program for missing data. J Stat Softw. 2011;45(7):1-47. doi:10.18637/jss.v045.i07

[30] Christensen RHB. A tutorial on fitting cumulative link mixed models with clmm2 from the ordinal package. The R Foundation; 2019. Accessed January 15, 2020. https://cran.r-project.org/web/packages/ordinal/vignettes/clmm2_tutorial.pdf

[31] Stone M, Dimofte C, Strong D, et al. Evaluating US smokers’ willingness to pay for different cigarette packaging designs before and after real-world exposure in a randomised trial. *Tob Control*. Published online March 1, 2022. doi:10.1136/tobaccocontrol-2021-057071 PMC11759659 35232793

