# Supplementary material for: Effect of Graphic Warning Labels on Cigarette Pack–Hiding Behavior Among Smokers: The CASA Randomized Clinical Trial
Source: JAMA Netw Open. 2022 Jun 2;5(6):e2214242. doi: 10.1001/jamanetworkopen.2022.14242 (PMC9164006; doi:10.1001/jamanetworkopen.2022.14242)
Supplement: Supplement 2. — eTable 1. Sample Characteristics by Randomization Group eTable 2. Ordinal Logistic Model of Weekly Assessments of Cigarette Pack-Hiding Behavior During Repackaging Intervention and After Return to Purchasing Their Usual US Pack eFigure 1. CASA Study Design eFigure 2. Images of Cigarette Packs Manufactured for the CASA Randomized Clinical Trial eFigure 3. Probability of Sometimes or Often “Placing the Pack Where Others Would Not See It” During the Intervention Period by Study Group, With 95% CIs eFigure 4. Patterns in Pack-Hiding Behavior by Study Group Assessed Weekly During the 3-Month Intervention and 8-Month Follow-up Periods [file jamanetwopen-e2214242-s002.pdf]

Supplementary Online Content

Pierce JP, Kealey S, Leas EC, et al. Effect of graphic warning labels on cigarette pack–hiding behavior among smokers: the CASA randomized clinical trial. *JAMA Netw Open*. 2022;5(6):e2214242. doi:10.1001/jamanetworkopen.2022.14242

eTable 1. Sample Characteristics by Randomization Group

eTable 2. Ordinal Logistic Model of Weekly Assessments of Cigarette Pack-Hiding Behavior During Repackaging Intervention and After Return to Purchasing Their Usual US Pack

eFigure 1. CASA Study Design

eFigure 2. Images of Cigarette Packs Manufactured for the CASA Randomized Clinical Trial

eFigure 3. Probability of Sometimes or Often "Placing the Pack Where Others Would Not See It" During the Intervention Period by Study Group, With 95% CIs

eFigure 4. Patterns in Pack-Hiding Behavior by Study Group Assessed Weekly During the 3-Month Intervention and 8-Month Follow-up Periods

This supplementary material has been provided by the authors to give readers additional information about their work.

© 2022 Pierce JP et al. *JAMA Network Open*.

eTable 1. Sample Characteristics by Randomization Group

Characteristic	Standard US N = 115	GWL Pack N = 117	Blank Pack N = 125	*P*-Value
Age at study entry	39.37 (11.61)	39.31 (12.18)	39.57 (11.88)	.98 [b]
Gender				.08 [a]
Male	44 (38.3%)	62 (53.0%)	56 (44.8%)	
Female	71 (61.7%)	55 (47.0%)	69 (55.2%)	
Race/Ethnicity				.88 [a]
Hispanic	10 (8.7%)	15 (12.8%)	15 (12.0%)	
Non-Hispanic White	80 (69.6%)	77 (65.8%)	86 (68.8%)	
Other non-Hispanic[#]	25 (21.8%)	25 (21.4%)	24 (19.2%)	
Education				.45 [a]
No Degree	62 (53.9%)	70 (59.8%)	77 (61.6%)	
College Degree	53 (46.1%)	47 (40.2%)	48 (38.4%)	
Income*				.25 [a]
Less than $49,999	49 (62.0%)	46 (54.8%)	48 (49.5%)	
$50,000 or more	30 (38.0%)	38 (45.2%)	49 (50.5%)	
Nicotine Dependence	3.84 (2.26)	3.68 (2.29)	3.90 (2.30)	.74 [b]
Last 7 days Cigarettes/day	13.03 (10.23)	11.85 (8.73)	12.86 (8.89)	.58 [b]
Serious Quit Attempt in past year	53 (46.1%)	48 (41.0%)	55 (44.0%)	.74 [a]
Smoke-Free Home	84 (73.0%)	88 (75.2%)	98 (78.4%)	.62 [a]
Family concern with smoking	56 (48.7%)	62 (53.0%)	66 (52.8%)	.76 [a]
Current Cigarette Brand				.89 [a]
Marlboro/Camel/American Spirit	98 (85.2%)	100 (85.5%)	109 (87.2%)	
Other	17 (14.8%)	17 (14.5%)	16 (12.8%)	
Appeal of Current Pack	3.51 (1.32)	3.85 (1.06)	3.74 (1.19)	.09 [b]
Feel loyal to current brand	84 (73.0%)	88 (75.2%)	97 (77.6%)	.71
Mental Health (K-6)	0.95 (0.67)	1.00 (0.75)	0.99 (0.78)	.85 [b]
Sensation Seeking	1.86 (0.65)	1.90 (0.61)	1.87 (0.74)	.87 [b]

Note. Data expressed as N(%) or Mean (SD).
[#]Other non-Hispanic including American Indian or Alaska Native (n=4), Asian (n=26) Pacific Islander (n=6), Black or African American (n=13), other (n=19), decline to answer (n=6)
*Income question was not asked of first 99 people in study
[a] Calculated using the ANOVA test.
[b] Calculated using the χ2 test.

© 2022 Pierce JP et al. *JAMA Network Open*.

eTable 2. Ordinal Logistic Model of Weekly Assessments of Cigarette Pack-Hiding Behavior During Repackaging Intervention and After Return to Purchasing Their Usual US Pack

Study Variables		Estimate	SE	z	p-value
Age		0.00	0.01	-0.04	0.970
Sex	Male	ref			
	Female	0.39	0.24	1.62	0.106
Race-ethnicity	Non-Hispanic White	ref			
	Hispanic	-0.84	0.39	-2.14	**0.032**
	Other Non-Hispanic	0.06	0.29	0.22	0.826
Nicotine Dependence		-0.06	0.05	-1.14	0.254
Baseline Appeal of Brand		0.31	0.10	3.06	**0.002**
Baseline tendency to conceal scale		0.52	0.35	1.47	0.143
Pack hiding prior to randomization		2.23	0.15	14.40	**<0.0001**
Study Arm effect	US pack	ref			
	GWL pack	0.40	0.96	0.42	0.674
	Blank pack	-2.18	0.98	-2.23	**0.026**
Intervention Timing					
	Initial Effect: Weeks 1-4	-0.46	0.26	-1.76	0.079
	Later Effect: Weeks 5-12	0.59	0.32	1.83	0.067
Study Period	Intervention	ref			
	Post-Intervention	-0.35	0.31	-1.15	0.250
Initial effect by Study Group	US pack	ref			
	GWL pack	1.51	0.37	4.14	**<0.0001**
	Blank pack	0.45	0.38	1.19	0.236
Later effects by Study Group	US pack	ref			
	GWL pack	0.12	0.46	0.26	0.798
	Blank pack	-0.28	0.48	-0.57	0.566
Post intervention by Study Group	US pack	ref			
	GWL pack	-2.00	0.44	-4.52	**<0.0001**
	Blank pack	0.81	0.48	1.69	0.090
**Interactions with Tendency to conceal scale**					
	on hiding during vs post intervention	0.17	0.15	1.09	0.276
	on hiding by Study arm: US pack	ref			
	GWL pack	-0.28	0.51	-0.54	0.592
	Blank pack	0.94	0.51	1.85	0.065
	on hiding during vs after intervention by study arm				
	US pack	ref			
	GWL pack	0.78	0.23	3.43	**0.001**
	Blank pack	-0.48	0.24	-1.98	**0.048**

Missing data were imputed (40 imputed data sets) and Rubin's Rule was used to pool estimates in Amelia in R software
Abbreviations: US =smoker's usual US cigarette pack; GWL=cigarette pack featuring 1 of 3 rotating images; Blank=cigarette pack devoid of any cigarette branding

© 2022 Pierce JP et al. *JAMA Network Open*.

eFigure 1. CASA Study Design

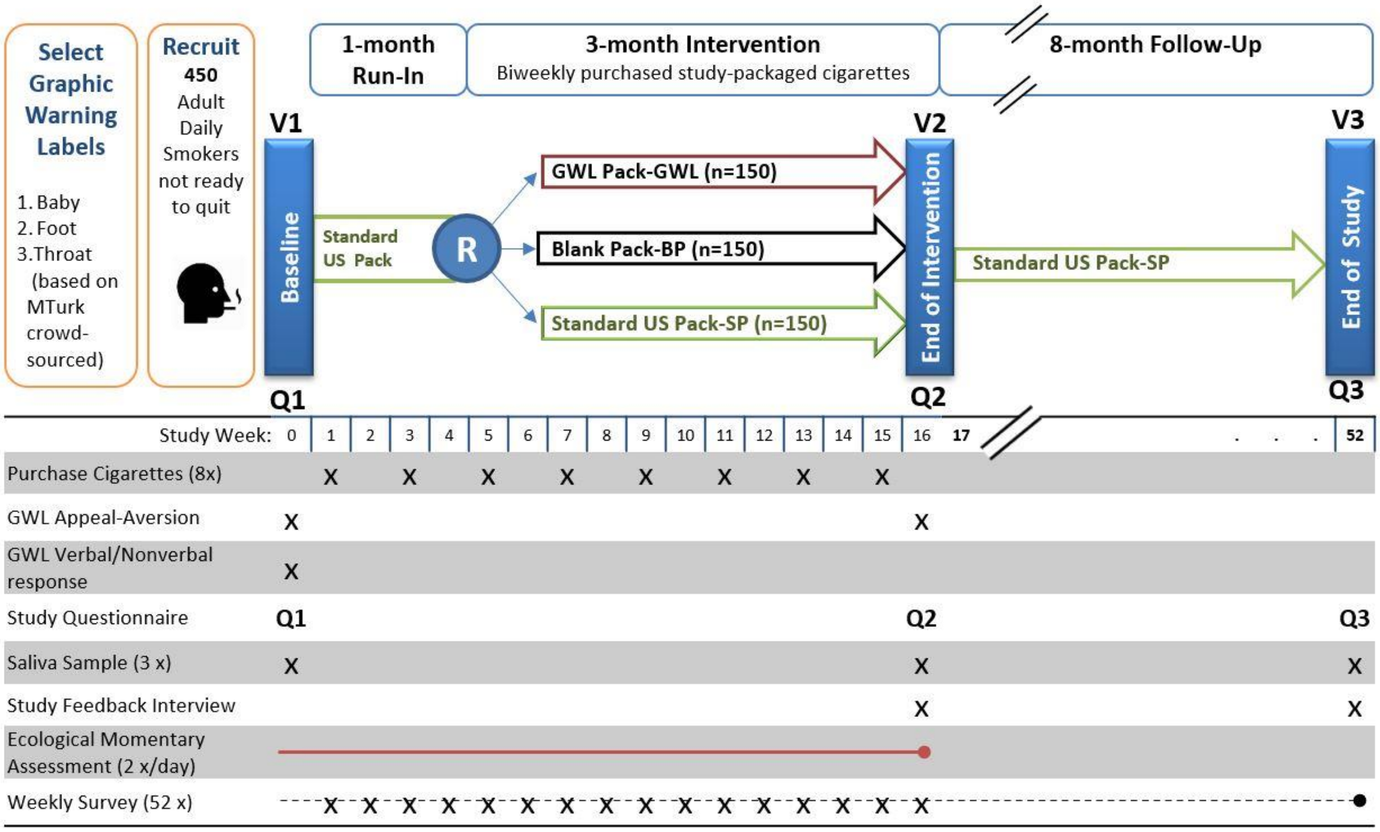

Reprinted from Pierce JP, Strong DR, Stone MD, et al. Real-world exposure to graphic warning labels on cigarette packages in US smokers: The CASA randomized trial protocol. *Contemp Clin Trials*. 2020;98:106152.

© 2022 Pierce JP et al. *JAMA Network Open*.

eFigure 2. Images of Cigarette Packs Manufactured for the CASA Randomized Clinical Trial

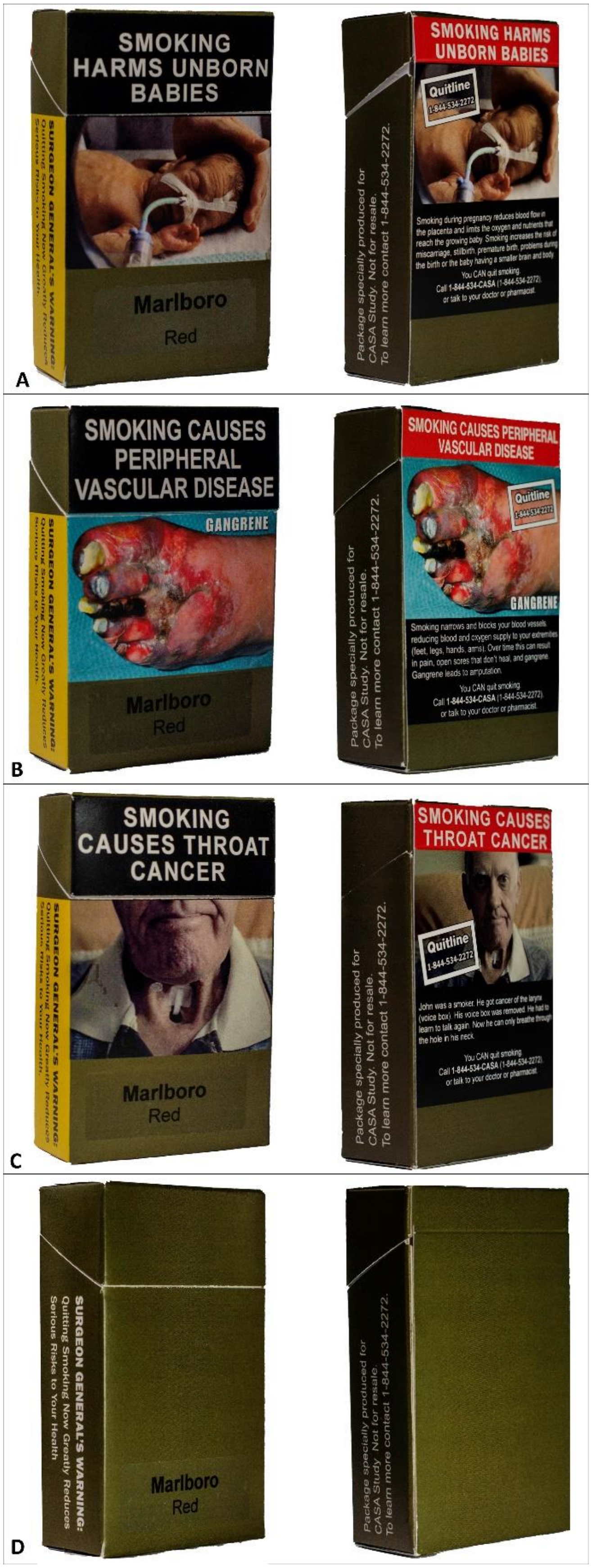

(A) Neonatal Baby*, (B) Foot Gangrene*, (C) Throat Cancer*, (D) Blank Pack.

Reprinted from Pierce JP, Strong DR, Stone MD, et al. Real-world exposure to graphic warning labels on cigarette packages in US smokers: The CASA randomized trial protocol. *Contemp Clin Trials*. 2020;98:106152.* © Commonwealth of Australia, used under license.

© 2022 Pierce JP et al. *JAMA Network Open*.

eFigure 3. Probability of Sometimes or Often "Placing the Pack Where Others Would Not See It" During the Intervention Period by Study Group, With 95% CIs

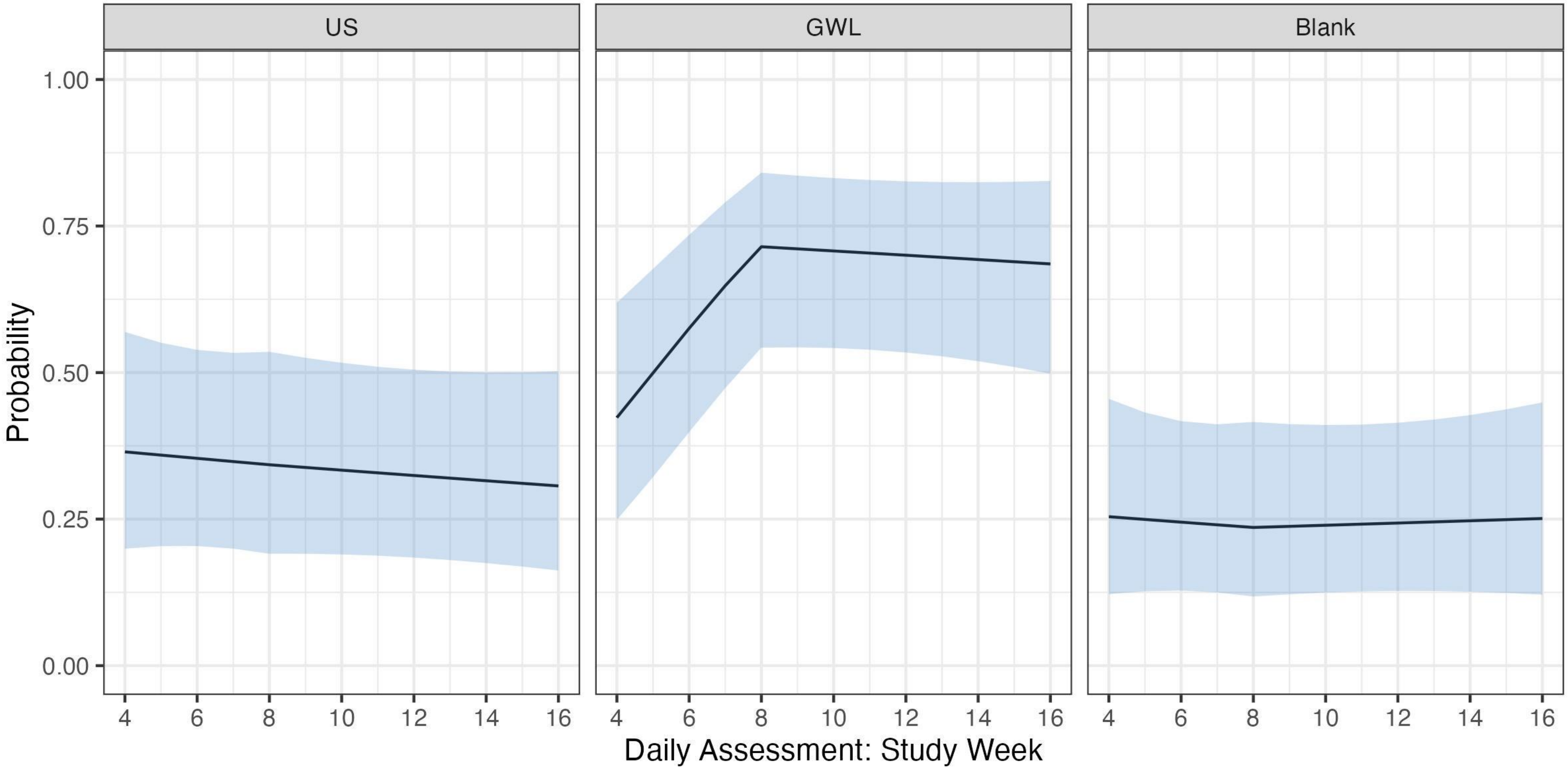

Abbreviations: US =smoker's usual US cigarette pack; GWL=cigarette pack featuring 1 of 3 rotating images; Blank=cigarette pack devoid of any cigarette branding

© 2022 Pierce JP et al. *JAMA Network Open*.

eFigure 4. Patterns in Pack-Hiding Behavior by Study Group Assessed Weekly During the 3-Month Intervention and 8-Month Follow-up Periods

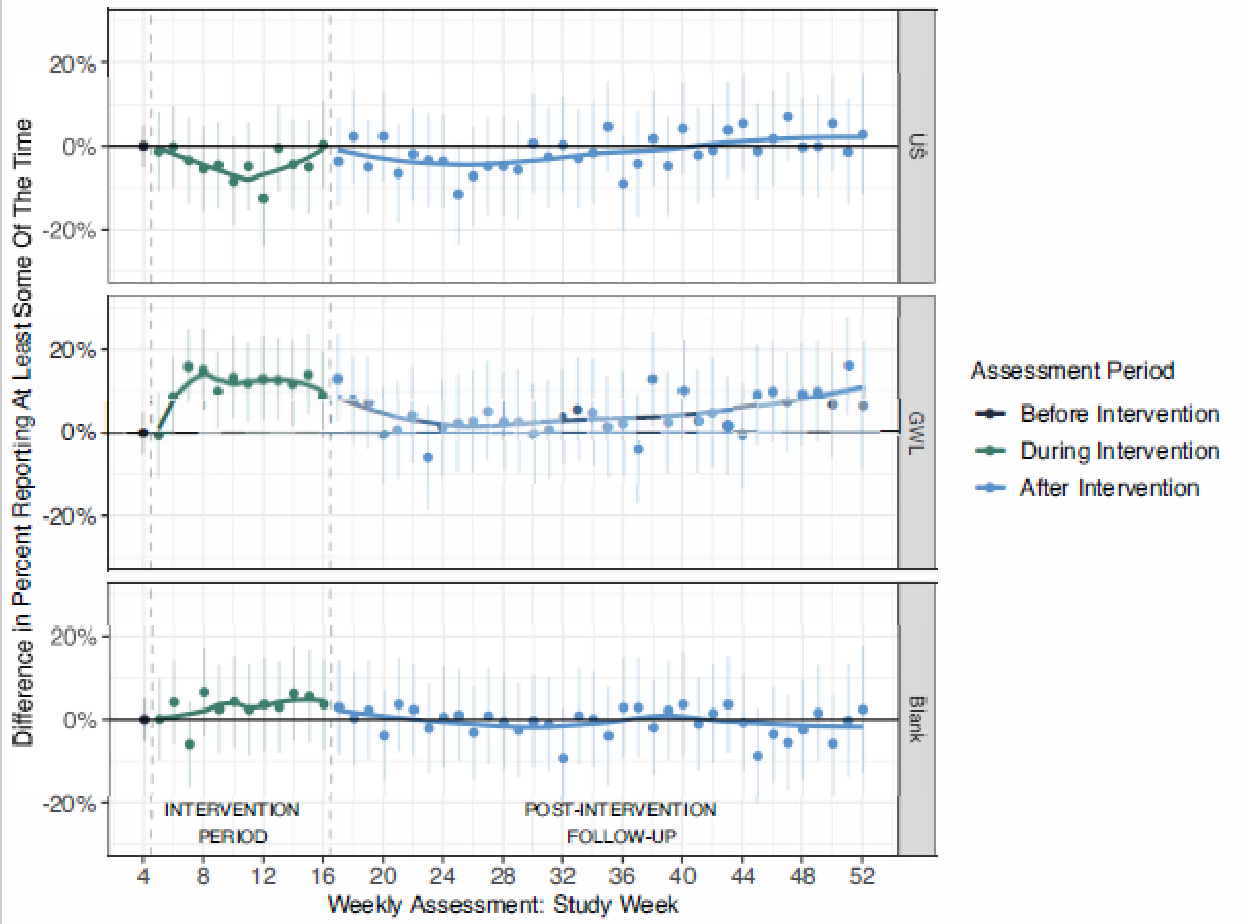

Abbreviations: US=smoker's usual US cigarette pack; GWL=cigarette pack featuring 1 of 3 rotating images; Blank=cigarette pack devoid of any cigarette branding

© 2022 Pierce JP et al. *JAMA Network Open*.
