# Supplementary material for: Effect of Graphic Warning Labels on Cigarette Pack–Hiding Behavior Among Smokers: The CASA Randomized Clinical Trial
Source: JAMA Netw Open. 2022 Jun 2;5(6):e2214242. doi: 10.1001/jamanetworkopen.2022.14242 (PMC9164006; doi:10.1001/jamanetworkopen.2022.14242)
Supplement: Supplement 3. — Data Sharing Statement [file jamanetwopen-e2214242-s00.pdf]

Data Sharing Statement

Pierce. Effect of Graphic Warning Labels on Cigarette Pack- Hiding Behavior Among Smokers. *JAMA Netw Open*. Published June 02, 2022. doi:10.1001/jamanetworkopen.2022.14242

Data
Data available: Yes
Data types: Deidentified participant data
How to access data: Data will be deposited with the UC San Diego Library, url not allocated yet
When available: With publication

Supporting Documents
Document types: None

Additional Information
Who can access the data: anyone requesting data
Types of analyses: for any purpose
Mechanisms of data availability: without investigator support
